# *Kappa*-opioid receptor gene (*OPRK1*) variations associated with opioid abstinence behaviors among chronic heroin users

**DOI:** 10.3389/fphar.2025.1714546

**Published:** 2025-11-28

**Authors:** Mark K. Greenwald, Catherine Demery, Tabitha E. H. Moses, Margit Burmeister

**Affiliations:** 1 Department of Psychiatry and Behavioral Neurosciences, Wayne State University School of Medicine, Detroit, MI, United States; 2 Department of Pharmacology, University of Michigan, Ann Arbor, MI, United States; 3 Michigan Neuroscience Institute and Department of Computational Medicine & Bioinformatics, University of Michigan, Ann Arbor, MI, United States

**Keywords:** OPRK1, kappa opioid receptor, heroin, abstinence, relapse, buprenorphine

## Abstract

**Introduction:**

Research suggests *kappa*-opioid receptors (KORs) modulate drug use and stress-related behaviors. While some findings indicate KORs could influence initial susceptibility to opioid use disorder (OUD), few studies have examined whether variations in the gene encoding the receptor (*OPRK1*) relate to clinically-relevant behavioral variation among current opioid users. This study examined whether *OPRK1* polymorphisms predicted opioid-abstinence phenotypes in three separate but conceptually-linked aims: (1) retrospective self-report of number of lifetime heroin-quit attempts at screening, (2) prospective assessment of opioid-abstinence initiation during a two-week buprenorphine (8 mg/day sublingual) outpatient stabilization period, and (3) prospective assessment of opioid lapse during a three-week buprenorphine dose-tapering outpatient period (4-mg/day, 2-mg/day and 0-mg/day during weeks 1-3, respectively).

**Methods:**

*OPRK1* genotype and opioid-abstinence phenotype data (urinalysis and self-report) were obtained from current regular heroin users. Genotype-phenotype analyses controlled for self-identified race and heroin-use duration.

**Results:**

*OPRK1* rs7817710 (intron) *T/T* homozygotes (*n *= 145) reported significantly more heroin-quit attempts than *G/T* heterozygotes (*n *= 86) or *G/G*-homozygotes (*n *= 35). During outpatient buprenorphine stabilization, *OPRK1* rs6989250 (intron) *C/C* homozygotes (*n *= 43) provided a significantly lower proportion of opioid-free urine samples than *G*-allele carriers (*n *= 7). During buprenorphine dose tapering, *OPRK1* rs3802281 (3’UTR) C-allele carriers (*n *= 21) and rs7817710 *G*-allele carriers (*n *= 11) lapsed to opioid use significantly more slowly than *T/T* homozygotes at either locus (*n *= 17 and *n *= 16, respectively). The rs3802281–rs7817710 haplotype block was associated with Experiment 1 binary phenotypes.

**Conclusion:**

These findings implicate *OPRK1* genetic variation in several opioid-abstinence phenotypes. These results, if replicated, could improve understanding of the course and treatment of OUD.

## 1 Introduction

Opioid use disorder (OUD) is a chronic, relapsing condition that is highly prevalent, disabling, and costly (Centers for Disease Control and Prevention, 2021; Degenhardt et al., 2019; Florence et al., 2021; Gomes et al., 2014; Humphreys et al., 2022; Joint Economic Committee Democrats, 2020; Kolodny et al., 2015). Gene × environment interactions are likely involved at all stages of OUD (Khokhar et al., 2010; Kreek et al., 2005a; Li and Burmeister, 2009). The idealized goal of using central nervous system or hepatic pharmacogenetic evidence to tailor OUD treatment for patients has proved elusive: thus far we lack compelling data on pivotal genes, biological pathways, and precise behavioral phenotypes necessary to achieve this objective.

Case-controlled studies that examined whether single nucleotide polymorphisms (SNPs) relate to OUD susceptibility have identified associations with multiple loci, including genes encoding the *mu*, *kappa,* and *delta* opioid receptors (*OPRM1*, *OPRK1,* and *OPRD1*, respectively) (Beer et al., 2013; Fudalej et al., 2016; Kreek et al., 2005b; Kumar et al., 2012; Levran et al., 2014a; Levran et al., 2014b; Levran et al., 2014c; Mayer et al., 1997; Yuferov et al., 2004). Most associations have not been subjected to a replication attempt, some failed replication, and others may be race-dependent or account for minimal variance. Furthermore, even if genetic variation partly explains vulnerability to OUD (Levran et al., 2008; Demery-Poulos and Chambers, 2021), this is less consequential for clinical practice after progression to OUD. Thus, pharmacogenetic studies of individuals with OUD that focus on phenotypes related to *drug use itself* (e.g., patterns and consequences of use) or *treatment response*, rather than susceptibility, are likely to have more direct relevance to clinical practice and outcomes.

A major impetus of phenotyping drug-use behaviors is targeting outcomes closely aligned with underlying neurobiological mechanisms of reinforcement, tolerance, and physical dependence (e.g., opioid receptor binding and signaling), or intermediate phenotypes such as reward motivation (Davis et al., 2008; Heitzeg et al., 2014), impulsivity (Kreek et al., 2005b; Moses et al., 2019), or stress reactivity (Levran et al., 2014b; Levran et al., 2014c; Xu et al., 2013). Using this approach, we previously observed that the *BDNF* ^66^Met allele, which affects BDNF secretion and neuroplasticity, was related to more “invested” drug-seeking behaviors (e.g., longer drug purchasing times) in regular heroin users (Greenwald et al., 2013). We also found that the *OPRM1* ^118^G allele, generally associated with reduced receptor expression (Wang et al., 2014a; Huang et al., 2012; Beyer et al., 2004), correlated with more lifetime adverse heroin-related consequences and quit attempts, and a higher likelihood of having sought treatment for heroin use in White regular heroin users (Woodcock et al., 2015a).

*Kappa*-opioid receptors (KORs) are functionally important because they coregulate, with *mu*-and *delta*-opioid receptors, tonic activity of endogenous opioid and dopamine systems (Yuferov et al., 2004; Darcq and Kieffer, 2018; Di Chiara and Imperato, 1988; Escobar et al., 2020; Spanagel et al., 1992). In the context of chronic drug use, KORs function as an anti-reward system that contributes to mood/stress dysregulation (Carlezon et al., 2009; Tejeda and Bonci, 2019; Zan et al., 2015) and drug use (Butelman et al., 2012; Wee and Koob, 2010; Xuei et al., 2006; Marchette et al., 2025). Accordingly, *OPRK1* genotypes have been associated with various facets of OUD (Kumar et al., 2012; Yuferov et al., 2004; Levran et al., 2008; Gerra et al., 2007; Yuanyuan et al., 2018). To illustrate, Wang et al. (2014b) reported that an *OPRK1* haplotype (rs10958350–rs7106778–rs12675595) was related to three opioid withdrawal signs (gooseflesh, yawning, restlessness) among Taiwanese patients in methadone treatment. Albonaim et al. (2017) observed that rs6985606 was associated with insomnia among Iranian methadone-maintained patients. Zhang et al. (2020) found that rs3802279 (*C/C*), rs3802281 (*T/T*), and rs963549 (*C/C*) genotypes were associated with lower methadone daily maintenance doses among Chinese heroin users. Yet, other studies have not identified reliable associations of *OPRK1* genotypes with opioid-related phenotypes (Gerra et al., 2014; Jones et al., 2016; Kibitov et al., 2016).

Clinical pharmacogenetic studies often employ relatively small sample sizes compared to vulnerability studies. One strategy for overcoming Type I error due to smaller sample sizes is to examine associations of hypothesized SNPs/haplotypes with an array of phenotypes that index a common construct, and to conduct experiments aimed at internal replication/extension of findings. The present series of three interrelated studies examined several *OPRK1* SNPs–candidates that were previously associated with initial vulnerability to OUD–for their relationship with opioid-abstinence behaviors: (1) retrospective self-report of lifetime heroin quit attempts, (2) prospective assessment of opioid abstinence initiation during outpatient stabilization on buprenorphine (a partial MOR agonist and KOR antagonist), and (3) prospective assessment of opioid lapse during a standardized outpatient buprenorphine dose taper. We hypothesized that *OPRK1* variation would correlate with opioid abstinence behaviors across these conditions.

## 2 Methods

### 2.1 Participants

The retrospective study (Experiment 1) used participant screening data obtained from four clinical studies (NCT00218309, NCT00218361, NCT00608504, and NCT00684840 registered on ClinicalTrials.gov) approved by Institutional Review Boards at Wayne State University and the University of Michigan and conducted in accordance with the Declaration of Helsinki. The prospective studies (Experiments 2 and 3) obtained data from participants who completed the latter three studies.

Participants were recruited via media advertisements and word-of-mouth referrals from the Detroit, Michigan metropolitan catchment area. Non-treatment seeking individuals (18–55 years of age) who used heroin regularly (at least weekly) and denied major medical or psychiatric disorders were invited for in-person screening. Participants were eligible to complete the in-person battery if they tested positive on the urine drug screen (UDS) for opioids (>300 ng/mL), negative for alcohol (<0.02%), and were cognitively intact (i.e., scored ≥80 on the Shipley Institute of Living Scale (Zac and hary, 1991)). Urine samples were not tested for oxycodone (not commonly misused in the Detroit metropolitan area) or fentanyl; the source studies concluded in 2012, prior to the emergence of fentanyl in the illicit drug supply.

### 2.2 Genotyping

Whole blood samples (6 mL per participant) were collected into EDTA tubes and DNA was extracted using the Oragene self-collection kit (Qiagen, Valencia, CA; formerly Gentra Puregene). The Golden Gate drug addiction Illumina panel (Hodgkinson et al., 2008) was used to genotype *OPRK1* rs6989250 (5′UTR), rs7817710 (intron) and rs6473797 (intron), and the Sequenom SNP MassARRAY iPLEX platform (Gabriel et al., 2009) was used to genotype *OPRK1* rs3802281 (3′UTR) and rs1051660 (5′UTR).

### 2.3 Phenotyping

In Experiment 1, heroin-use phenotypes were derived from a standardized self-report substance use history battery (Drug History and Use Questionnaire [DHUQ; available on request]). All participants provided onsite UDS and were asked about history and patterns of use (e.g., duration of use, quit attempts, adverse consequences of use) for heroin and other major substances. To probe the substance specificity of this relationship, we explored associations of these *OPRK1* SNPs with lifetime consequences and quit attempts related to other substances often used by primary heroin users, i.e., tobacco, alcohol, cannabis and cocaine.

To explore mechanisms underlying *OPRK1* phenotypic associations, we examined two other domains of phenotypes measured in a subset of Experiment 1 participants: naturalistic drug-purchasing behaviors and consumption from the Drug Purchasing and Use Questionnaire (DPUQ; (Greenwald et al., 2013; Roddy and Greenwald, 2009; Roddy et al., 2011); and drug use-specific impulsivity from the Impulsive Relapse Questionnaire (IRQ (Moses et al., 2019; Stoltman et al., 2015). The IRQ has five subscales measuring different dimensions of drug-use impulsivity; higher scores on each IRQ subscale indicate higher impulsivity.

Analyses in Experiments 2 and 3 focused on UDS and self-reported opioid abstinence. A subset of participants from Experiment 1 qualified for laboratory pharmacology studies and these participants gave separate informed consent and entered into Experiment 2. Briefly, participants in Experiment 2 had to meet DSM-IV criteria for current Opioid Dependence, lack other severe Drug Dependence for other substances except nicotine, lack serious psychiatric conditions (e.g., psychosis, bipolar disorder, major depression that was not substance induced), and lack chronic medical conditions (e.g., cardiovascular, pulmonary, neurological, hepatic, renal, systemic) or taking medications for these conditions.

As part of Experiment 2, opioid abstinence was measured via UDS and self-report 3 times/week over a 2-week outpatient stabilization on sublingual buprenorphine (8 mg/day). Following outpatient buprenorphine stabilization, participants entered a laboratory-based inpatient study (∼14–20 days). A further subset of the participants who completed Experiment 2 underwent Experiment 3, which involved a standardized 3-week outpatient buprenorphine dose taper (4 mg/day, 2 mg/day, and 0 mg/day during weeks 1, two and 3, respectively) combined with an opioid abstinence-contingent money incentive, with thrice-weekly UDS and self-report of drug use (Greenwald, 2008; Woodcock et al., 2015b).

### 2.4 Data analyses

Calculations for Hardy-Weinberg equilibrium (HWE) were conducted with raw allelic frequencies for the overall (Experiment 1) sample using Haploview software (Barrett et al., 2005). Using raw genotype frequencies, we calculated SNP-pairwise linkage disequilibrium (LD) values of *D*′, likelihood odds ratio (LOD) and *r* ^2^ using the SNPtools Excel add-in (Chen et al., 2009) and Haploview software, with *p*-values generated using SNPstats (Solé et al., 2006). Haplotype blocks were defined by the Four Gamete Rule (Menyhart et al., 2021). Associations between haplotypes and binary phenotype states (Table 1) were evaluated using Haploview software to determine case/control χ^2^ and corresponding *p*-values, with haplotype phases inferred using a standard EM algorithm; odds ratios and corresponding *p*-values were determined using a logistic regression model adjusted for race (SNPstats (Solé et al., 2006)). Multiple testing corrections were performed with False Discovery Rate = 5% (Menyhart et al., 2021).

**TABLE 1:** *OPRK1* linkage disequilibrium results for overall sample *D’*: normalized difference between observed and expected haplotype frequencies. LOD: likelihood odds ratio (a measure of confidence in *D*′).

Locus 1	Locus 2	*D*’	LOD	*r* ^2^	*p*-value
rs3802281	rs7817710	0.96	37.08	0.610	<0.0001
rs3802281	rs6473797	0.37	3.33	0.071	<0.0001
rs3802281	rs6989250	0.68	2.96	0.066	<0.0001
rs7817710	rs6473797	0.62	6.21	0.136	<0.0001
rs7817710	rs6989250	0.85	5.91	0.143	<0.0001
rs6473797	rs6989250	0.44	0.60	0.014	0.024

For genotype-phenotype association analyses, due to smaller cell sizes, minor allele carriers were often grouped together and compared with major allele homozygotes. Chi-square tests for assessing independence of each SNP from self-reported race (Black or White) were conducted using the collapsed groups (minor allele carriers vs. major allele homozygotes) except for rs6473797 (as both homozygotes were equally frequent).

Data were first screened for bivariate correlations between genotype groups and phenotypes. For significant associations, stepwise multiple linear regression or analysis of covariance (ANCOVA) was conducted, controlling for self-reported race and duration of regular heroin use. Sex was examined but was not significant in any analyses and is not discussed further.

All descriptive data are presented as mean ± one standard deviation. Continuous variables that were initially non-normally distributed were log_10_ (raw value + 1)-transformed for analyses (e.g., heroin quit attempts, duration of heroin use, and proportion of outpatient urine samples positive for illicit opioid use). All analyses were conducted with SPSS v.30 and used the criterion of *p* < 0.05 to reject the null hypothesis.

## 3 Results

### 3.1 OPRK1 genotype characteristics

*OPRK1* genotype frequencies did not significantly deviate from HWE for rs7817710, rs6989250, rs3802281, or rs6473797 in the overall sample nor when separated by race (Supplementary Tables 1a–1d). *OPRK1* rs1051660 genotype frequencies significantly deviated from HWE (Supplementary Tables 1e), so this genotype was not considered further. There were no sex differences in genotype frequency of any SNP.

*OPRK1* rs7817710, rs6989250, and rs3802281 genotype frequencies were significantly associated with race, χ^2^ (1) = 24.81 (*p* < 0.001), 5.63 (*p* < 0.02), and 33.19 (*p* < 0.001), respectively, as was rs6473797, χ^2^ (2) = 24.85 (*p* < 0.001). Supplementary Tables 2a–2c indicate racial distributions of collapsed genotype groups for rs7817710, rs6989250, and rs3802281. Using a minor allele dominant model, minor allele carriers were contrasted with major allele homozygotes, except for rs6473797, which had equal allele frequencies.

**FIGURE 1:**
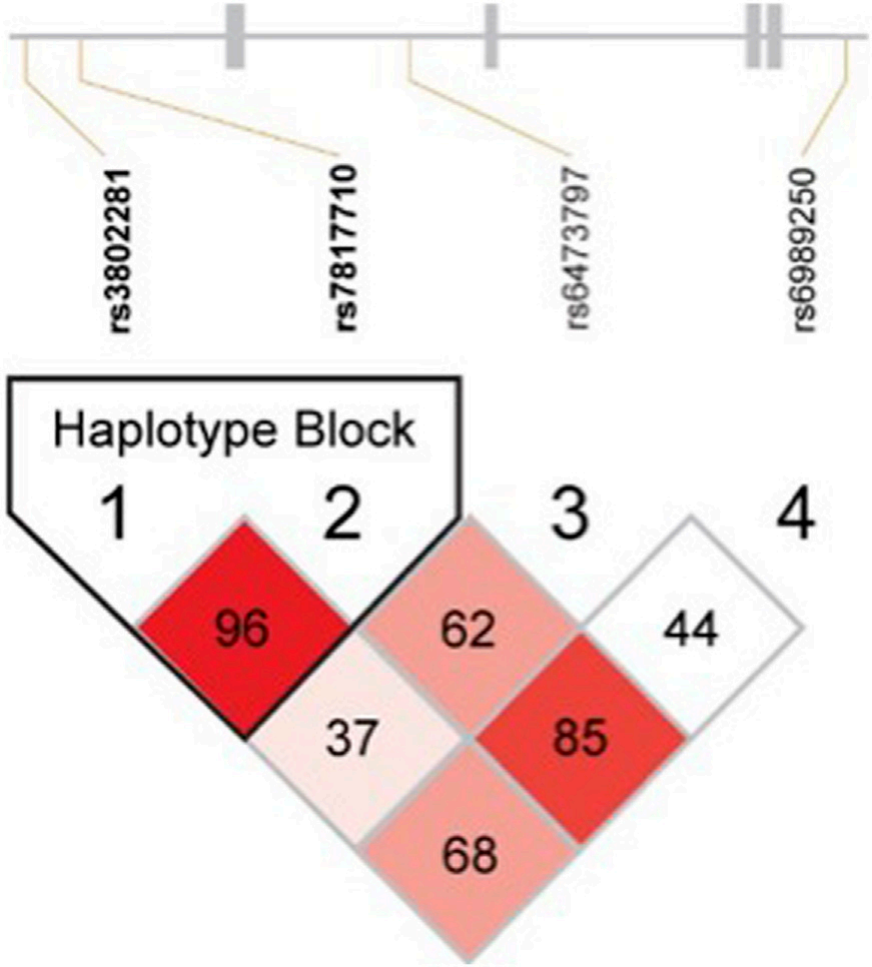
Linkage disequilibrium results for all *OPRK1* SNPs in Hardy-Weinberg Equilibrium (rs3802281, rs7817710, rs6473797, and rs6989250) in the overall sample. Numbers represent D′ value (normalized difference between observed and expected haplotype frequencies), with colors varying by D′ and likelihood odds ratio (LOD) as follows: *D*’<1, LOD<2 = White; *D*’<1, LOD≥2 = pink-to-red shades. Blocks were defined by the Four Gamete Rule (Wang et al., 2002).

These four *OPRK1* SNPs were in strong LD with one another in the Experiment 1 overall sample (Figure 1; Table 2) and when separated by race (Supplementary Figure 1; Supplementary Table 3), except that rs3802281 and rs6473797 were not in LD in the White participant sample, as measured by the standardized disequilibrium value (*D*′) determined using Haploview software (Barrett et al., 2005) (LD results for Experiment 2 and 3 overall samples are reported in Supplementary Table 4). A haplotype block, defined using the Four Gamete Rule (Wang et al., 2002), between rs3802281 and rs7817710 was identified in the overall sample (Figure 1) and Black cohort (Supplementary Figure 1a), in contrast to the haplotype block between rs7817710, rs6473797, and rs6989250 identified in the White cohort (Supplementary Figure 1b). Association of haplotypes in the overall sample (adjusted for race) with binary phenotypes are reported by experiment.

**TABLE 2:** Demographic and substance use characteristics across experimental subgroups.

Variables	Exp. 1	Exp. 2	Exp. 3
Demographics	(n = 191)	(n = 52)	(n = 38)
Race (% Black, White)	57.9%, 42.1%	50%, 50%	53.8%, 46.2%
Sex (% male)	76.3%	78.8%	76.9%
Age (years)	43.6 ± 9.1	41.7 ± 9.2	41.1 ± 10.0
Heroin	(n = 191)	(n = 52)	(n = 38)
Duration of use (years)	20.6 ± 11.9	18.0 ± 11.4	17.1 ± 12.2
Current injection use (%)	63.9%	62.7%	60.5%
Use days in past month	28.6 ± 3.6	29.2 ± 1.7	29.1 ± 1.9
Avg. Uses/day (past week)	3.8 ± 2.7	4.8 ± 5.5	3.6 ± 3.5
Total consequences (max = 18)	7.5 ± 4.5	7.5 ± 4.3	7.9 ± 4.3
Heroin overdose ever (%)	33.2%	30.8%	28.2%
Quit attempts	10.3 ± 18.2	9.3 ± 15.2	7.4 ± 8.7
Heroin treatment ever sought (%)	76.7%	75.0%	69.2%
Tobacco	(n = 181)	(n = 51)	(n = 32)
Use days in past month	26.3 ± 9.1	26.6 ± 9.1	25.7 ± 10.2
Cigarettes/day	15.0 ± 9.9	14.0 ± 7.9	12.8 ± 7.4
Total consequences (max = 16)	4.9 ± 4.3	5.3 ± 4.3	4.3 ± 3.6
Quit attempts	2.7 ± 4.3	3.5 ± 5.0	3.0 ± 4.9
Alcohol	(n = 184)	(n = 51)	(n = 32)
Use days in past month	3.5 ± 6.7	2.6 ± 5.5	2.8 ± 6.2
Total consequences (max = 20)	2.8 ± 4.3	3.1 ± 4.3	2.5 ± 4.1
Quit attempts	2.8 ± 3.4	1.0 ± 2.2	1.1 ± 2.5
Marijuana	(n = 160)	(n = 48)	(n = 32)
Use days in past month	3.5 ± 7.3	2.4 ± 4.0	2.7 ± 4.3
Total consequences (max = 22)	2.9 ± 3.9	2.5 ± 3.1	2.0 ± 2.6
Quit attempts	1.3 ± 2.8	1.1 ± 2.2	1.0 ± 2.0
Cocaine	(n = 70)	(n = 28)	(n = 32)
Use days in past month	5.4 ± 8.1	3.4 ± 6.3	3.3 ± 6.8
Total consequences (max = 18)	3.0 ± 3.8	2.1 ± 2.7	2.3 ± 3.1
Quit attempts	6.2 ± 17.1	1.4 ± 2.3	1.5 ± 2.3

### 3.2 Phenotypic characteristics across experimental subgroups

Table 2 compares demographic and substance-use characteristics for successive subgroups of participants who completed Experiments 1, 2 and 3; there were no significant differences across subgroups. The average participant was a 42-year-old male with a high-school education, who had used heroin for nearly 2 decades, currently used on a daily basis, tended to inject (more than insufflate) heroin, endorsed 8 of 21 lifetime heroin-use consequences, and reported 10 lifetime attempts to quit heroin. Most participants smoked cigarettes (92% endorsed past-month use, typically daily), and were intermittent users of alcohol, cannabis, and cocaine.

Importantly, the three opioid abstinence phenotypes examined across Experiments 1, 2 and 3 were not significantly correlated with one another (Supplementary Table 5).

### 3.3 Experiment 1: Retrospective association of OPRK1 genotype and heroin-use phenotypes

#### 3.3.1 Heroin-quit attempts

We examined relationships between *OPRK1* genotypes and self-reported lifetime heroin quit attempts (log_10_-transformed). *OPRK1* rs7817710 (*T/T* vs. *G*-allele carriers) had a significant bivariate (Kendall *tau*) association with quit attempts (*r* = 0.202, *p* = 0.001), as did rs3802281 genotype (*T/T* vs. *C*-allele carriers; *r* = 0.125, *p* = 0.045). In stepwise linear regression analysis, the latter SNP was not significantly related to heroin quit attempts after controlling for race and duration of heroin use.

**FIGURE 2:**
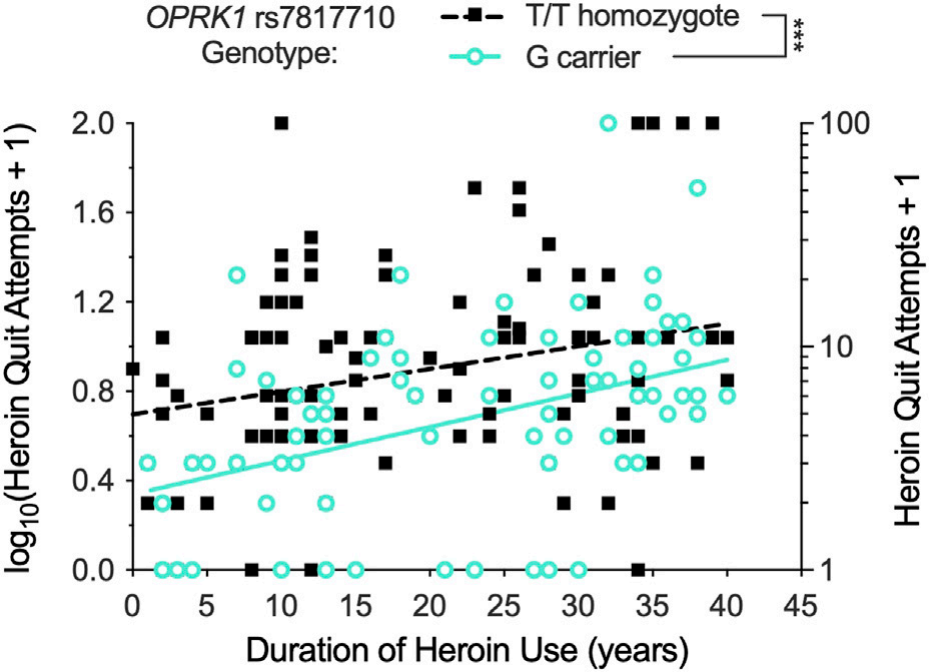
Distribution of Exp. 1 participants’ lifetime heroin quit attempts (raw values + 1; right Y-axis) and log10 transform of these data (left Y-axis), and relationship of quit attempts to duration of regular heroin use (illustrated in actual years, but analyzed using log_10_ data). Lifetime heroin quit attempts significantly increased with duration of heroin use (positive slope of regression lines), and *OPRK1* rs7817710 *T/T* homozygotes reported significantly more lifetime quit attempts than *G*-allele carriers (difference in Y-intercept of the parallel regression lines). ***p < 0.001.

Figure 2 presents results from stepwise multiple linear regression analysis of log_10_ heroin quit attempts, which found significant effects of all three predictors: log_10_ duration of heroin use (*β* = 0.395, *t* = 5.23, *p* < 0.001, Δ*r* ^2^ = 0.093), *OPRK1* rs7817710 binary genotype (*β* = 0.233, *t* = 3.14, *p* = 0.002, Δ*r* ^2^ = 0.081), and race (White > Black; *β* = 0.160, *t* = 1.98, *p* = 0.049, Δ*r* ^2^ = 0.019), together accounting for 17.9% (adjusted *r* ^2^) of variance in the outcome (overall *F* [3,173] = 13.57, *p* < 0.001). Participants with *OPRK1* rs7817710 *T/T* genotype reported significantly more heroin quit attempts (*n* = 106; M [±1 SD] = 0.90 [0.43]) than *G*-allele carriers (*n* = 73; M [±1 SD] = 0.66 [0.43]).

Indeed, ANCOVA found heroin quit attempts were a function of *OPRK1* rs7817710 genotype (*F* (1,176) = 11.30, *p* < 0.001, *η* ^*2*^ = 0.060), controlling for a significant effect of duration of heroin use (*F* (1,176) = 28.90, *p* < 0.001, *η* ^*2*^ = 0.141), with no significant race main effect (*p* = 0.115) or genotype × race interaction (*p* = 0.254). We next evaluated whether rs3802281/rs7807710 haplotype was associated with >10 heroin quit attempts. The two most frequent haplotypes, T/T (χ^2^ = 8.52, *p* = 0.006) and C/G (χ^2^ = 8.90, *p* = 0.006), were significantly related to the binary phenotype of >10 quit attempts. The C/G haplotype was protective, indicated by a significantly lower (race-adjusted) odds ratio compared to the T/T haplotype: 0.43 (95% CI: 0.24–0.79; *p* = 0.016), as shown in Table 3 and Figure 5.

**TABLE 3:** Haplotype associations with experiment 1 phenotypes in overall sample, *n* = 182 patients genotyped at both loci; rs3802281/rs7817710, *D*’ = 0.96, *r* ^2^ = 0.61.

Phenotype	rs3802281	rs7817710	HT frequency	χ^b^ (*p*-value^a^)	% HT Affected (cases/total HT)	% Affected with HT (cases/total cases)	OR^a^ (95% CI)	OR *p*-value^a^ ^,^ ^b^
>10 heroin quit attempts	T	T	0.652	8.523 (0.006)	38.51	75.40	1.00	n/a
	C	G	0.248	8.898 (0.006)	20.73	15.40	0.43 (0.24–0.79)	0.016
	C	T	0.094	0.008 (0.930)	32.68	9.21	0.76 (0.35–1.65)	0.490
History of heroin overdose	T	T	0.652	7.702 (0.009)	37.70	75.00	1.00	n/a
	C	G	0.248	18.047 (0.0006)	14.96	11.29	0.41 (0.21–0.78)	0.016
	C	T	0.094	1.618 (0.244)	42.25	12.10	1.49 (0.70–3.20)	0.400

HT, haplotype; OR, odds ratio relative to the most frequent haplotype (TT).

^a^ *p*-values adjusted with False Discovery Rate (FDR) = 5%.

^b^Adjusted by race.

#### 3.3.2 Drug-use pattern

After controlling for race and duration of heroin use, there were no significant effects of *OPRK1* rs7817710 *T/T* genotype (vs. *G*-allele carriers) on DPUQ phenotypes of round-trip heroin purchasing times, unit purchase amount, number of weekly purchases, nor daily number of $10-equivalent bags of heroin consumed.

#### 3.3.3 Drug-use impulsivity

We examined whether *OPRK1* genotypes differed in drug-use impulsivity, which might contribute to (unproductive) quit attempts. There were significant Kendall *tau* correlations of rs7817710 genotype with IRQ scores on Capacity for Delay (*r* = 0.153, *p* = 0.044) and Speed (*r* = 0.165, *p* = 0.033), with *T/T* genotype exhibiting higher impulsivity scores than *G*-allele carriers. Using ANCOVA that controlled for race and duration of heroin use, rs7817710 genotype effect remained significant for IRQ Capacity for Delay scores (M [±1 SD] = 26.4 [5.9] vs. 24.2 [7.3] for *T/T* vs. *G*-allele carriers, respectively), *F* (1,118) = 4.86, *p* = 0.030, *η* ^*2*^ = 0.040, but was no longer significant for IRQ Speed, *F* (1,118) = 1.18, *p* = 0.280. Other IRQ subscale scores did not differ by genotype.

#### 3.3.4 Lifetime drug-use consequences

Participants with *OPRK1* rs7817710 *T/T* genotype reported significantly higher likelihood of a lifetime heroin overdose than *G*-allele carriers (44.4% vs. 20.3%, respectively; *χ* ^2^ = 11.34, *p* < 0.001), as did participants with *OPRK1* rs3802281 *T*/*T* genotype compared to *C*-allele carriers (43.7% vs. 23.8%, respectively; *χ* ^2^ = 8.39, *p* = 0.004). Indeed, rs3802281/rs7807710 haplotype was significantly associated with a history of heroin overdose (*T*/*T*: χ^2^ = 7.70, *p* = 0.009; *C*/*G*: χ^2^ = 18.05, *p* = 0.0006). The *C*/*G* haplotype conferred a significantly lower odds ratio of 0.41 relative to the *T*/*T* haplotype (95% CI: 0.21–0.78; *p* = 0.016; Table 3; Figure 5). After controlling for race and duration of heroin use, there was no significant effect of *OPRK1* rs7817710 *T/T* genotype (vs. *G*-allele carriers) on total lifetime heroin-use consequences.

We explored specificity of the genotype × drug-use phenotype findings by using ANCOVA to examine subgroups of participants who endorsed progressing (lifetime) to regular use of other substances. Controlling for race and age, participants with *OPRK1* rs7817710 *T/T* genotype (vs. *G*-allele carriers) reported significantly more adverse consequences from regularly using alcohol, *F* (1,54) = 11.59, *p* = 0.001, *η* ^*2*^ = 0.188, with non-significant effects for tobacco, *F* (1,158) = 3.73, *p* = 0.055, cannabis, *F* (1,116) = 3.07, *p* = 0.082, and cocaine, *F* (1,51) = 3.33, *p* = 0.074. Among participants who progressed to regular use of these other substances, participants with *OPRK1* rs7817710 *T/T* genotype (vs. *G*-allele carriers) reported significantly more quit-attempts for alcohol, *F* (1,53) = 4.72, *p* = 0.035, *η* ^*2*^ = 0.088, but not tobacco, *F* (1,156) = 0.068, *p* = 0.79, cannabis, *F* (1,115) = 2.23, *p* = 0.14, or cocaine, *F* (1,51) = 0.52, *p* = 0.23.

### 3.4 Experiment 2: Prospective association of OPRK1 genotype and opioid abstinence initiation during buprenorphine stabilization

**FIGURE 3:**
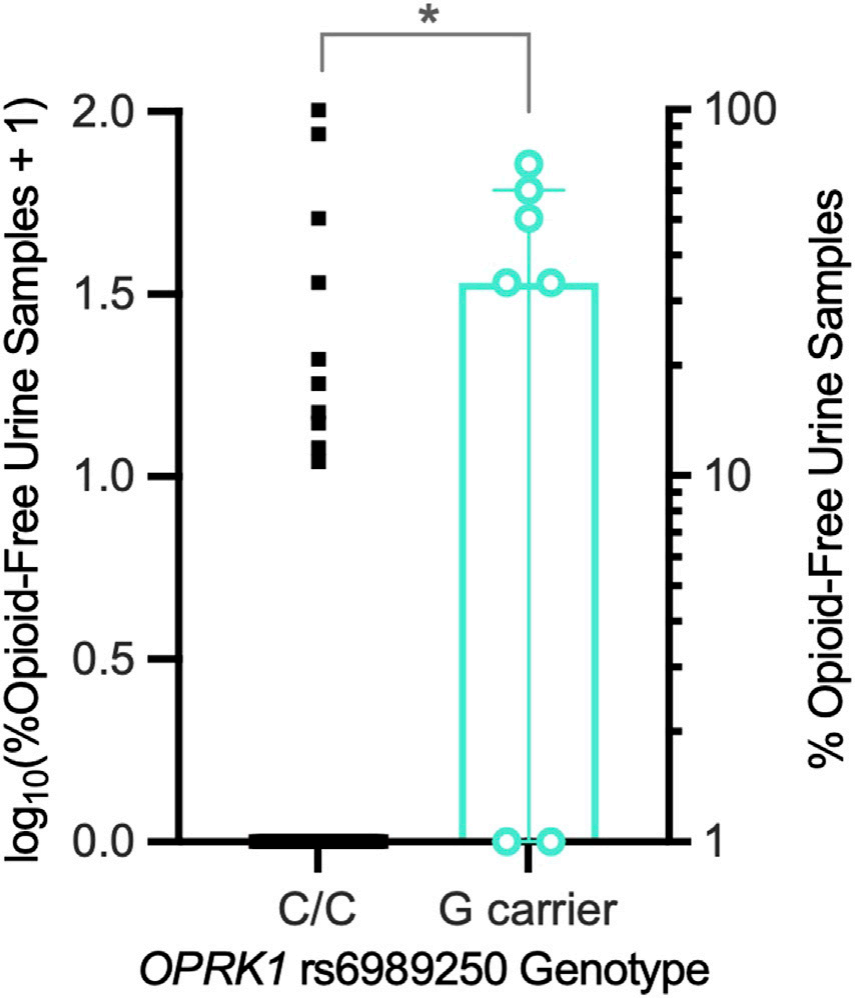
Exp. 2 participants’ proportions of illicit opioid-free urine samples during a 2-week outpatient period of stabilization on buprenorphine (8-mg/day SL). *OPRK1* rs6989250 *G*-allele carriers (*n* = 7) provided a significantly higher mean (horizontal line) proportion of opioid-free urine samples than *C/C* homozygotes (*n* = 45), controlling for other factors. *p < 0.05.

Next, we examined the relationship between *OPRK1* genotype and opioid abstinence initiation in a buprenorphine-maintained subsample of the Experiment 1 group (Table 2). *OPRK1* rs6989250 genotype significantly predicted the log_10_ proportion of illicit opioid-free urine samples during buprenorphine stabilization (*β* = −0.378; adjusted *r* ^2^ = 0.122, *t* = −2.62, *p* = 0.012), controlling for race, duration of heroin use, heroin quit attempts, and other *OPRK1* genotypes examined here (all latter variables were non-significant). Figure 3 illustrates that *G*-allele carriers (*n* = 7) provided significantly more opioid-negative urine samples than did *C/C* homozygotes (*n* = 45). Corresponding with the lack of effect of SNPs rs3802281 and rs7817710 on the proportion of opioid-free urine drug samples independently, Table 4 and Figure 5 show that there was no significant association of rs3802281/rs7817710 haplotype with the binary phenotype of no abstinence (i.e., zero urine samples negative for illicit opioids).

**TABLE 4:** Haplotype association with Experiment 2 binary phenotype in overall sample, n = 44 patients genotyped at both loci; rs3802281/rs7817710,*D*’ = 1.00,*r* ^2^= 0.56.

Phenotype	rs3802281	rs7817710	HT frequency	χ^b^(*p*-value^a^)	% HT Affected (cases/total HT)	% Affected with HT (cases/total cases)	OR^a^(95% CI)	OR*p*-value^a^ ^,^ ^b^
Zero abstinence during buprenorphine stabilization	T	T	0.676	2.341 (0.312)	24.64	56.67	1.00	n/a
	C	G	0.207	1.586 (0.312)	40.57	28.67	0.43 (0.12–1.47)	0.360
	C	T	0.116	0.411 (0.522)	37.29	14.67	0.61 (0.16–2.38)	0.480

HT, haplotype; OR, odds ratio relative to the most frequent haplotype (TT).

^a^ *p*-values adjusted with FDR, 5%.

^b^ Adjusted by race.

### 3.5 Experiment 3: Prospective association of OPRK1 genotype and heroin lapse during buprenorphine dose tapering

**FIGURE 4:**
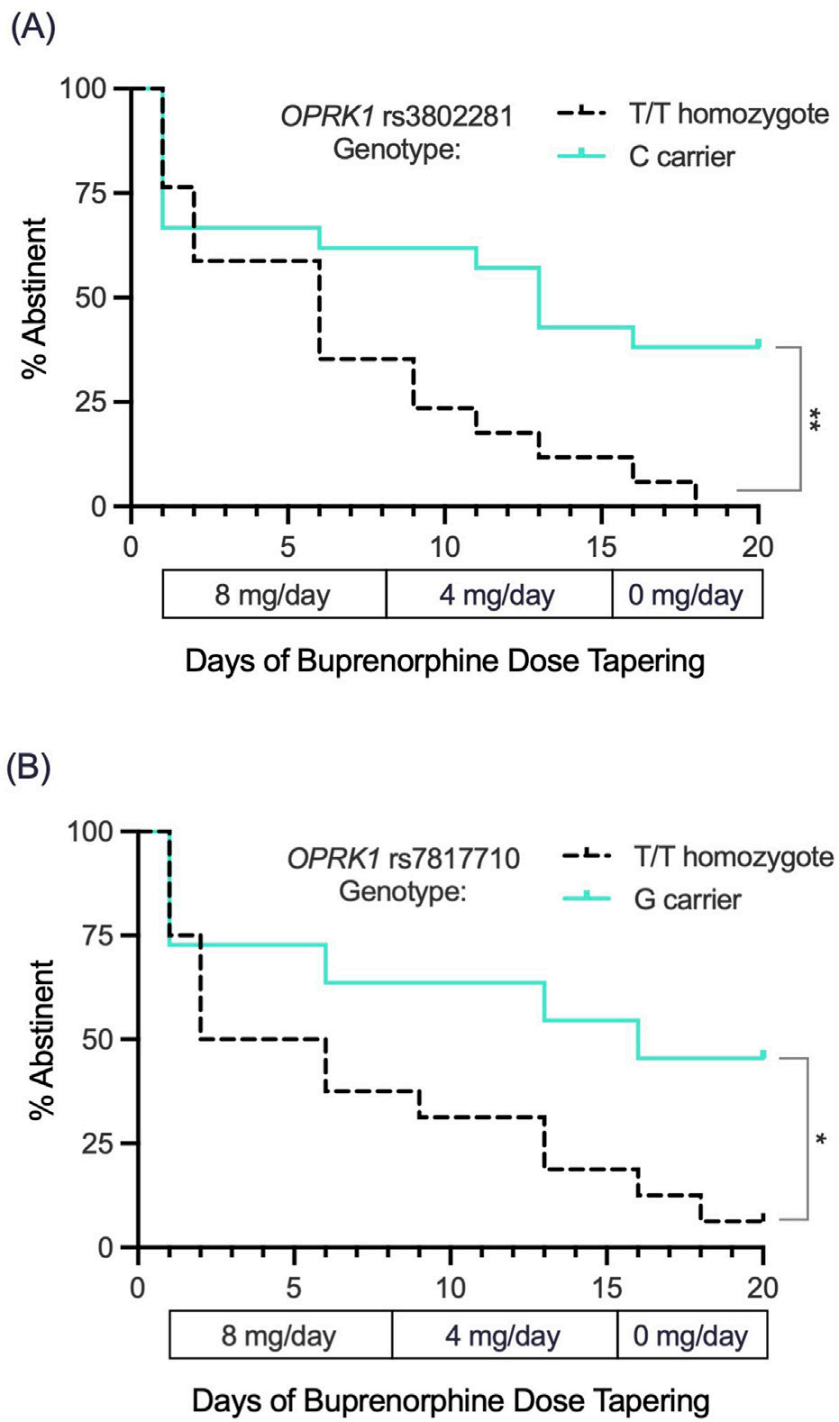
Exp. 3 survival curve analysis of lapse to opioid use (based on thrice-weekly urine drug screen and self-report) during a standardized 3-week buprenorphine dose taper (4-mg/day, 2-mg/day and 0-mg/day SL during weeks n1, 2 and 3, respectively). **(A)** *OPRK1* rs3802281 *C*-allele carriers (*n* = 21) lapsed to opioid use significantly more slowly than *T/T* homozygotes (*n* = 17). **(B)** *OPRK1* rs7817710 *G*-allele carriers (*n* = 11) also lapsed significantly more slowly to illicit opioid use compared to *T/T* homozygotes (*n* = 16). *p < 0.05, **p < 0.01.

**FIGURE 5:**
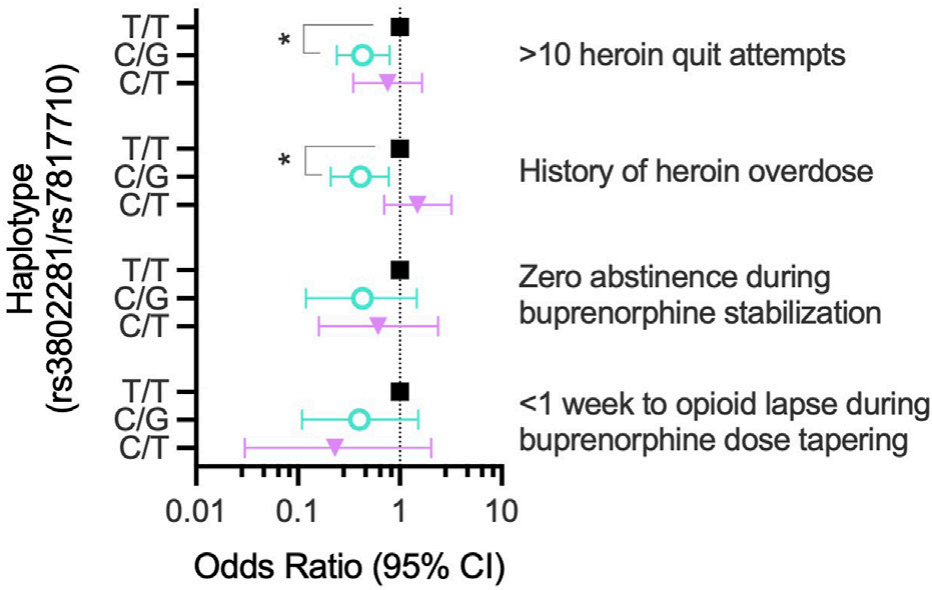
*OPRK1* haplotype (rs3802281/rs7807710) association with binary opioid-abstinence phenotypes in experiments 1-3 (right of graph). Within each phenotype, the odds ratio for a given haplotype (adjusted for race) is shown relative to the most frequent haplotype (T/T, Black squares). **p < 0.05.

We followed 38 participants throughout their 3-week outpatient buprenorphine dose taper (Table 2). Survival curve analysis found that both *OPRK1* rs3802281 and rs7817710 genotypes were significantly associated with lapse from heroin abstinence during this period. Figure 4A shows that rs3802281 *C*-allele carriers (*n* = 21) lapsed more slowly to heroin use than *T/T* homozygotes (*n* = 17), log rank χ^2^ = 7.20, *p* = 0.007. In addition, *OPRK1* rs7817710 G-allele carriers (*n* = 11) lapsed from abstinence significantly slower than T/T homozygotes (*n* = 16), log rank χ^2^ = 4.38, *p* = 0.036 (Figure 4B). The T/T haplotype of these loci was non-significantly associated with <7 days to illicit opioid lapse: χ^2^ = 5.39, *p* = 0.060 (Table 5). As with experiment 1 binary phenotypes, the C/G haplotype was associated with a lower odds ratio of 0.40 relative to the T/T haplotype, yet this did not reach significance (95% CI: 0.11–1.52, *p* = 0.159; Figure 5).

**TABLE 5:** Haplotype association with Experiment 3 binary phenotype in overall sample,*n*= 27 patients genotyped at both loci; rs3802281/rs7817710,*D*’ = 1.00,*r* ^2^= 0.70.

Phenotype	rs3802281	rs7817710	HT frequency	χ^b^(*p*-value^a^)	% HT Affected (cases/total HT)	% Affected with HT (cases/total cases)	OR^a^(95% CI)	OR*p*-value^a^ ^,^ ^b^
<7 days to opioid lapse during buprenorphine dose tapering	T	T	0.606	5.390 (0.060)	40.00	47.06	1.00	n/a
	C	G	0.306	2.615 (0.159)	66.34	39.41	0.40 (0.11–1.52)	0.190
	C	T	0.088	1.894 (0.169)	79.31	13.53	0.23 (0.03–2.04)	0.190

HT, haplotype; OR, odds ratio relative to the most frequent haplotype (TT).

^a^ *p*-values adjusted with FDR, 5%.

^b^ Adjusted by race.

Stepwise multiple linear regression analysis found that *OPRK1* rs3802281 genotype accounted for 18% of variance in number of days to heroin lapse, (*β* = −0.461; adjusted *r* ^2^ = 0.180, *t* = −2.55, *p* = 0.018), controlling for race, duration of heroin use, heroin quit attempts (Exp. 1), proportion of illicit opioid-free UDS during buprenorphine stabilization (Exp. 2), and other *OPRK1* genotypes (all of which were not significantly related to opioid lapse).

## 4 Discussion

KORs have been implicated in the compulsive use of opioids and other substances (Yuferov et al., 2004; Darcq and Kieffer, 2018; Butelman et al., 2012; Wee and Koob, 2010; Marchette et al., 2025; Bazov et al., 2018). We found that *OPRK1* variants were significantly related to three clinically relevant opioid use/abstinence phenotypes. Specifically, when controlling for race and duration of regular heroin use, *OPRK1* polymorphisms explained substantial variance in: (1) retrospective self-reports of lifetime heroin quit attempts (rs781770, *r* ^2^ = 0.081), (2) opioid abstinence initiation during buprenorphine stabilization (rs698250, *r* ^2^ = 0.142), and (3) opioid lapse during buprenorphine dose tapering (rs3802281, *r* ^2^ = 0.180). Meanwhile, rs6473797 was not significantly associated with any opioid abstinence phenotypes evaluated herein.

Interestingly, no single genotype was (after multivariate control) significantly associated across these three phenotypes, which is perhaps unsurprising because these phenotypes did not significantly correlate with each other (i.e., evidence of discriminant validity). Nonetheless, the haplotype block of rs3802281/rs7817710 was associated with binary phenotypes in Experiment 1 (and marginally in Experiment 3), but not Experiment 2, in line with the findings for these SNPs independently.

Experiment 1 focused on *OPRK1* variation in lifetime heroin quit attempts. Among these out-of-treatment regular heroin users, this phenotype offers an estimate of prior unsuccessful efforts to become abstinent from heroin. Repetitive quit attempts reflect the chronic, relapsing nature of OUD. After controlling for race (White participants endorsed more quit-attempts than Black participants) and duration of heroin use (longer duration associated with more quit-attempts), participants with rs7817710 *T/T* genotype reported significantly (≈36%) more heroin quit attempts than *G*-allele carriers. Furthermore, there was a significant association between rs3802281/rs7817710 haplotype and reporting >10 heroin quit attempts, with the C/G haplotype being protective (OR 0.43) compared to the most frequent T/T haplotype.

The quit-attempt phenotype resembles one DSM-5 diagnostic criterion for OUD, “unsuccessful efforts or desire to cut back or control opioid use” (American Psychiatric Association, 2013), and more quit attempts could be interpreted as a problem of self-regulation. Thus, we examined whether *OPRK1* variation was related to self-reported drug-use impulsivity. After controlling for race and duration of heroin use, *OPRK1* rs7817710 *T/T* homozygotes exhibited higher drug-use impulsivity on the IRQ Capacity for Delay subscale. Moreover, both rs7817710 and rs3802281 genotypes and the combined (rs3802281/rs7817710) haplotype were significantly associated with having experienced any heroin overdose, with the C/G haplotype again being protective (OR 0.41) compared to the most frequent T/T haplotype. These findings suggest that *OPRK1* variation could modulate self-regulation, changing the incidence of negative outcomes including overdose and repeated unproductive quit attempts. In a study of opioid users (Wang et al., 2014b), rs7817710 *T*-allele carriers experienced more severe bone and joint pain during opioid withdrawal than *G*-allele homozygotes. The present study found that rs7817710 *T/T* homozygotes reported more lifetime heroin quit attempts. Taken together, a plausible working hypothesis is that *T*-allele carriers could experience certain adverse effects when trying to discontinue opioids, leading to multiple unsuccessful abstinence attempts and greater return to use. Indeed, genetic modulation of sensitivity to aversive symptoms during attempted opioid abstinence is another mechanism that could drive relapses, consistent with the role of KOR signaling in mediating negative reinforcement during the cycle of addiction (Darcq and Kieffer, 2018; Butelman et al., 2012; Bruijnzeel, 2009; Koob, 2015; Crowley and Kash, 2015; Koob, 2013).

Experiment 2 focused on the prospective analysis of *OPRK1* variation in illicit opioid abstinence initiation during the initial 2 weeks of outpatient stabilization on buprenorphine. Moderate doses of buprenorphine, such as used here (8 mg/day), are often effective for suppressing withdrawal symptoms while abstaining from opioid use, but are less effective than higher buprenorphine doses for blocking opioid use (Greenwald et al., 2014). Moderate-dose buprenorphine stabilization offers an experimental means to examine genetic variation in propensity to opioid use, independent of opioid withdrawal, which we previously found to be minimal in this same population (Greenwald et al., 2024). Under these conditions, and after controlling for race and lifetime duration of regular heroin use, *OPRK1* rs6989250 (located in the 5′UTR regulatory region and thus could influence gene expression) was associated with opioid abstinence initiation during buprenorphine stabilization: *G*-allele carriers (who are significantly more likely to be Black) provided significantly more opioid-negative urine samples than *C/C* homozygotes. It is not known whether *OPRK1* variation might interact with buprenorphine’s pharmacodynamic profile (*mu*-receptor partial agonist and KOR antagonist) to modulate endogenous opioid signaling and behavior. To our knowledge, there is only one other study of *OPRK1* rs6989250 related to drug use in which the *G*-allele (vs. *C*-homozygous) was associated with greater stress-reactivity and cocaine relapse among Black participants (Xu et al., 2013). Taken together, these findings underscore a broad role of *OPRK1* in addiction and treatment response (Butelman et al., 2012).

Experiment 3 focused on prospective analysis of *OPRK1* variation in illicit opioid lapse following an inpatient period of buprenorphine 8 mg/day. We examined speed of return to opioid use (i.e., days to illicit opioid lapse) during a 3-week outpatient buprenorphine dose taper with opioid abstinence-contingent money reinforcement (Woodcock et al., 2015b). This rate of buprenorphine dose tapering aligns with prior research and clinical practice (Dunn et al., 2011) and, using this approach, we demonstrated that the opioid abstinence contingency lengthens and increases variability in time to opioid lapse among non-treatment seeking participants (Greenwald, 2008). Both *OPRK1* rs3802281 and rs7817710 genotypes were significantly associated with return to heroin use during buprenorphine dose tapering: *C*-allele and *G*-allele carriers, respectively, lapsed more slowly to heroin use than *T/T* homozygotes, even after controlling for race, duration of heroin use, heroin quit attempts, and opioid abstinence during initial buprenorphine stabilization. The T/T haplotype rs3802281/rs7817710 (vs. the C/G haplotype) association with <7 days to illicit opioid lapse did not reach significance, likely due to the smaller sample size for this analysis. We cannot rule out alternative explanations that this genotype influenced sensitivity to the opioid abstinence behavioral contingency (i.e., non-drug reward) or its interaction with buprenorphine dose. Given the complex nature of OUD and its treatment, additional studies are needed to disentangle these mechanisms.

The present study have several limitations. First, participants were current heroin users who were not in treatment and not actively looking to quit using heroin. They had tried previously to quit using heroin; thus, by definition, these prior quit attempts were unsuccessful. As such, these findings can be considered in the larger framework of people with OUD, but we are careful not to extrapolate them to individuals who are actively seeking treatment. Second, this is a candidate gene study, based on *a priori* assumptions about *OPRK1* function. We recognize that: (a) our assumptions could be incorrect; (b) the present study does not prove these variants are causally related to observed differences; and (c) because two *OPRK1* SNPs studied (rs6473797 and rs1051660) were not significantly associated with phenotypes nor were in Hardy-Weinberg disequilibrium and not considered, respectively, we do not know the specific loci related to *OPRK1* putative influence. Third, some drug-use variables were self-reported, which may introduce recall bias; however, use of UDS data (Experiments 2 and 3) partly offsets this concern. Fourth, we used self-reported race rather than ancestral informative markers, although these measures were highly correlated in our prior analysis of *mu*-opioid receptor variation in this participant pool (Woodcock et al., 2015a). Fifth, successively smaller sample sizes in this study lead to reduced statistical power, particularly for experiment 3, genotype rs7817710. We tried to minimize this concern by developing *a priori* hypotheses to target analyses. Sixth, due to restricted sample size, we did not explore possible epistatic interactions of *OPRK1* with variants in genes for prodynorphin (*PDYN*, endogenous ligand at KORs) or *OPRM1*, which could better address the molecular neurobiological complexity underlying these phenotypes.

Despite these limitations, this conceptually integrated series of experiments supports a role of KORs in relation to opioid use and buprenorphine exposure. *OPRK1* variants were significantly associated with several (retrospective and prospective) opioid use/abstinence phenotypes. Notably, these phenotypes were statistically independent of one another, suggesting that they reflect distinct facets of opioid use/abstinence. This is likely not only due to temporal factors, but also because the two prospective phenotypes may be due to different neurobehavioral processes that underlie abstinence initiation vs. remaining abstinent. Nonetheless, we report evidence for the association of an *OPRK1* haplotype block (rs3802281/rs7817710) with various opioid-abstinence phenotypes from experiments 1 and 3, indicating that variation in this genomic region may have implications for multiple aspects of opioid abstinence. We noted evidence that supports a role for KORs in compulsive use of substances and regulating negative mood states, response to stress, and opioid withdrawal. It is unsurprising that KOR antagonists are being investigated as potential treatments for stress-related disorders including prevention of drug relapse (Carlezon et al., 2009; Butelman and Kreek, 2017; Greenwald, 2018; Jacobson et al., 2020; Redila and Chavkin, 2008).

In conclusion, these preliminary data demonstrate the utility of studying individual differences in opioid use and treatment responses. Initiating abstinence and preventing relapse are key clinical targets in OUD treatment; these *OPRK1* variants could be useful markers of buprenorphine treatment response. Future studies should (1) continue to refine the phenotype assessment, (2) examine mechanisms underlying these effects (e.g., impulsivity, opioid withdrawal or stress sensitivity), (3) extend analysis to epistasis with *PDYN* and *OPRM1*, and (4) expand the sample size for larger-scale testing of hypotheses related to abstinence initiation and protection against relapse.

## Data Availability

Data and variable-level metadata are available at: https://github.com/Mark-Greenwald-WSU/OPRK1-Heroin-Data
