# Supplementary material for: Kappa-opioid receptor gene (OPRK1) variations associated with opioid abstinence behaviors among chronic heroin users
Source: Front Pharmacol. 2025 Nov 28;16:1714546. doi: 10.3389/fphar.2025.1714546 (PMC12698606; doi:10.3389/fphar.2025.1714546)

**Supplemental Table 1 – Raw genotype and allele frequencies in the overall (Exp. 1) sample**

**Supp. Table 1a** *OPRK1* rs7817710

| rs7817710 (N) | *G/G* | *G/T* | *T/T* | *G* allele | *T* allele | HWE *p* |
| --- | --- | --- | --- | --- | --- | --- |
| Black (105) | 15  (14.3%) | 44  (41.9%) | 46  (43.8%) | 74  (35.2%) | 136  (64.8%) | 0.497 |
| White (77) | 1  (1.3%) | 14  (18.2%) | 62  (80.5%) | 16  (10.4%) | 138  (89.6%) | >0.999 |
| Overall (182) | 16  (8.8%) | 58  (31.9%) | 108  (59.3%) | 90  (24.7%) | 274  (75.3%) | 0.083 |

**Supp. Table 1b** *OPRK1* rs6989250

| rs6989250 (N) | *G/G* | *G/C* | *C/C* | *G* allele | *C* allele | HWE *p* |
| --- | --- | --- | --- | --- | --- | --- |
| Black (110) | 0  (0.0%) | 20  (18.2%) | 90  (81.8%) | 20  (9.1%) | 200  (90.9%) | >0.999 |
| White (79) | 0  (0.0%) | 5  (6.3%) | 74  (93.7%) | 5  (3.2%) | 153  (96.8%) | >0.999 |
| Overall (189) | 0  (0.0%) | 25 (13.2%) | 164  (86.8%) | 25  (6.6%) | 353  (93.4%) | >0.999 |

**Supp. Table 1c** *OPRK1* rs3802281

| rs3802281 (N) | *C/C* | *C/T* | *T/T* | *C* allele | *T* allele | HWE *p* |
| --- | --- | --- | --- | --- | --- | --- |
| Black (109) | 25  (22.9%) | 53  (48.6%) | 31  (28.4%) | 103  (47.2%) | 115  (52.8%) | 0.910 |
| White (79) | 2  (2.5%) | 21  (26.6%) | 56  (70.9%) | 25  (15.8%) | 133  (84.2%) | >0.999 |
| Overall (188) | 27  (14.4%) | 74  (39.4%) | 87  (46.3%) | 128  (34.0%) | 248  (66.0%) | 0.118 |

**Supp. Table 1d** *OPRK1* rs6473797

| rs6473797 (N) | *A/A* | *A/G* | *G/G* | *A* allele | *G* allele | HWE *p* |
| --- | --- | --- | --- | --- | --- | --- |
| Black (107) | 16  (15.0%) | 53  (49.5%) | 38  (35.5%) | 85  (39.7%) | 129  (60.3%) | 0.918 |
| White (79) | 32  (40.5%) | 40  (50.6%) | 7  (8.9%) | 104  (65.8%) | 54  (34.2%) | 0.386 |
| Overall (186) | 48  (25.8%) | 93  (50.0%) | 45  (24.2%) | 189  (50.8%) | 183  (49.2%) | >0.999 |

**Supp. Table 1e** *OPRK1* rs1051660

| rs1051660 (N) | *A/A* | *A/C* | *C/C* | *A* allele | *C* allele | HWE *p* |
| --- | --- | --- | --- | --- | --- | --- |
| Black (105) | 72  (69.2%) | 12  (11.5%) | 20  (19.2%) | 156  (75.0%) | 52  (25.0%) | <0.0001 |
| White (77) | 43  (57.3%) | 13  (17.3%) | 19  (25.3%) | 99  (66.0%) | 51  (34.0%) | <0.0001 |
| Overall (182) | 115  (64.3%) | 25  (13.0%) | 39  (21.8%) | 25  (71.2%) | 103  (28.8%) | <0.0001 |

**Supplemental Table 2 – Collapsed genotype groups**

**Supp. Table 2a** *OPRK1* rs7817710

| rs7817710 (N) | *G*-carrier | *T/T* |
| --- | --- | --- |
| Black (105) | 59 (56.2%) | 46 (43.8%) |
| White (77) | 15 (19.5%) | 62 (80.5%) |
| Overall (182) | 74 (40.7%) | 108 (59.3%) |

**Supp. Table 2b** *OPRK1* rs6989250

| rs6989250 (N) | *G*-carrier | *C/C* |
| --- | --- | --- |
| Black (126) | 20 (18.2%) | 90 (81.8%) |
| White (84) | 5 (6.3%) | 74 (93.7%) |
| Overall (210) | 25 (13.2%) | 164 (86.8%) |

**Supp. Table 2c** *OPRK1* rs3802281

| rs3802281 (N) | *C*-carrier | *T/T* |
| --- | --- | --- |
| Black (109) | 78 (71.6%) | 31 (28.4%) |
| White (79) | 23 (29.1%) | 56 (70.9%) |
| Overall (188) | 101 (53.7%) | 87 (46.3%) |

**Supplemental Table 3 – Linkage disequilibrium results by race**

*D*’ = normalized difference between observed and expected haplotype frequencies; LOD = likelihood odds ratio (a measure of confidence in *D*’)

**Supp. Table 3a** Linkage disequilibrium results for Black sample

| **Locus 1** | **Locus 2** | ***D*’** | **LOD** | ***r*^2^** | ***p*-value** |
| --- | --- | --- | --- | --- | --- |
| rs3802281 | rs7817710 | 1.00 | 24.14 | 0.63 | <0.0001 |
| rs3802281 | rs6473797 | 0.22 | 0.70 | 0.03 | 0.012 |
| rs3802281 | rs6989250 | 0.69 | 1.41 | 0.06 | <0.001 |
| rs7817710 | rs6473797 | 0.35 | 1.08 | 0.05 | 0.002 |
| rs7817710 | rs6989250 | 0.77 | 2.54 | 0.11 | <0.0001 |
| rs6473797 | rs6989250 | 0.69 | 2.52 | 0.08 | <0.0001 |

**Supplemental Table 3b** Linkage disequilibrium results for White sample

| **Locus 1** | **Locus 2** | ***D*’** | **LOD** | ***r*^2^** | ***p*-value** |
| --- | --- | --- | --- | --- | --- |
| rs3802281 | rs7817710 | 0.84 | 7.40 | 0.44 | <0.0001 |
| rs3802281 | rs6473797 | 0.22 | 0.19 | 0.02 | 0.112 |
| rs3802281 | rs6989250 | 0.42 | 0.39 | 0.03 | 0.028 |
| rs7817710 | rs6473797 | 1.00 | 4.59 | 0.24 | <.0001 |
| rs7817710 | rs6989250 | 1.00 | 2.22 | 0.18 | <.0001 |
| rs6473797 | rs6989250 | 1.00 | 1.18 | 0.06 | 0.002 |

**Supplemental Table 4 – Linkage disequilibrium results in Experiment 2 and 3 cohorts**

*D*’ = normalized difference between observed and expected haplotype frequencies; LOD = likelihood odds ratio (a measure of confidence in *D*’)

**Supp. Table 4a** Linkage disequilibrium results for Experiment 2 cohort

| **Locus 1** | **Locus 2** | ***D*’** | **LOD** | ***r*^2^** | ***p*-value** |
| --- | --- | --- | --- | --- | --- |
| rs3802281 | rs7817710 | 1.00 | 7.84 | 0.56 | <0.0001 |
| rs3802281 | rs6473797 | 0.22 | 0.44 | 0.03 | 0.068 |
| rs3802281 | rs6989250 | 0.54 | 0.74 | 0.05 | 0.021 |
| rs7817710 | rs6473797 | 0.63 | 1.64 | 0.16 | <0.001 |
| rs7817710 | rs6989250 | 1.00 | 2.52 | 0.21 | <0.0001 |
| rs6473797 | rs6989250 | 0.26 | 0.04 | 0.00 | 0.516 |

**Supp. Table 4b** Linkage disequilibrium results for Experiment 3 cohort

| **Locus 1** | **Locus 2** | ***D*’** | **LOD** | ***r*^2^** | ***p*-value** |
| --- | --- | --- | --- | --- | --- |
| rs3802281 | rs7817710 | 1.00 | 6.61 | 0.70 | <0.0001 |
| rs3802281 | rs6473797 | 0.41 | 1.09 | 0.13 | 0.004 |
| rs3802281 | rs6989250 | 0.46 | 0.39 | 0.04 | 0.089 |
| rs7817710 | rs6473797 | 0.59 | 1.55 | 0.21 | 0.002 |
| rs7817710 | rs6989250 | 1.00 | 1.88 | 0.23 | <0.0001 |
| rs6473797 | rs6989250 | 0.42 | 0.13 | 0.02 | 0.252 |

**Supplemental Table 5 – Pearson correlations (*r* and *p* values) among principal phenotypes in Experiments 1, 2 and 3**

|  | Heroin quit-attempts (Exp. 1) | % Opioid abstinence (Exp. 2) | Days to opioid lapse (Exp. 3) |
| --- | --- | --- | --- |
| Heroin quit-attempts | ----- | ----- | ----- |
| % Opioid abstinence | -0.225 (.09)  [*n*=58] | ----- | ----- |
| Days to opioid lapse | 0.010 (.95)  [*n*=39] | 0.124 (.45)  [*n*=40] | ----- |

**Supplemental Figure 1** Linkage disequilibrium results by race

Linkage disequilibrium results for all SNPs in Hardy-Weinberg Equilibrium (rs3802281, rs7817710, rs6473797, and rs6989250) generated using Haploview software. Numbers represent *D*’ value, with colors varying by *D*’ and LOD as follows: *D*’<1, LOD<2=White; *D*’<1, LOD≥2=pink-to-red shades; *D*’=1, LOD<2=blue; *D*’=1, LOD≥2=dark red. Blocks were defined by the Four Gamete Rule.

**Supp. Figure 1a** Linkage disequilibrium in Black cohort


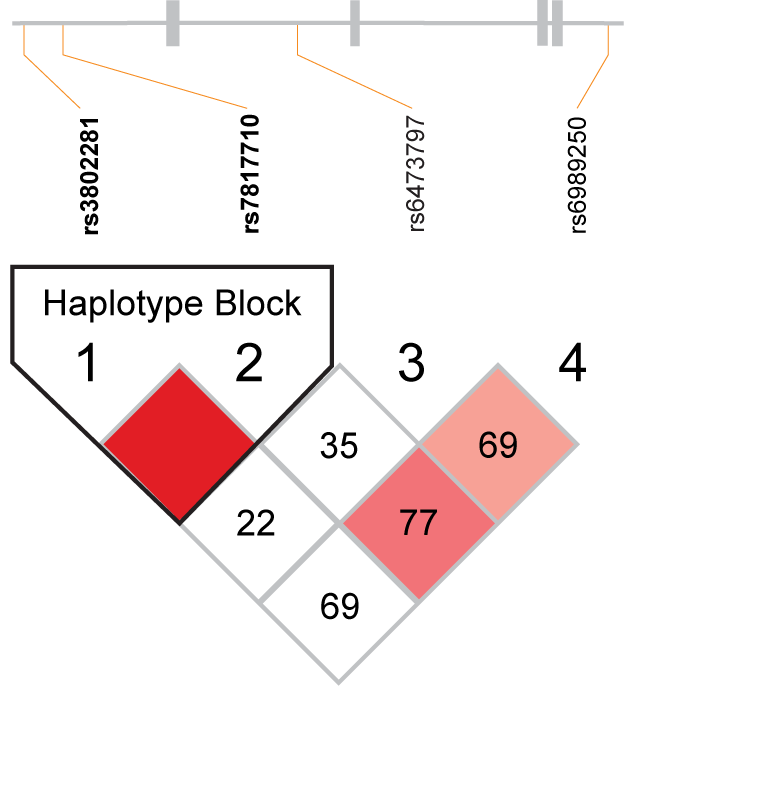


**Supp. Figure 1b** Linkage disequilibrium in White cohort


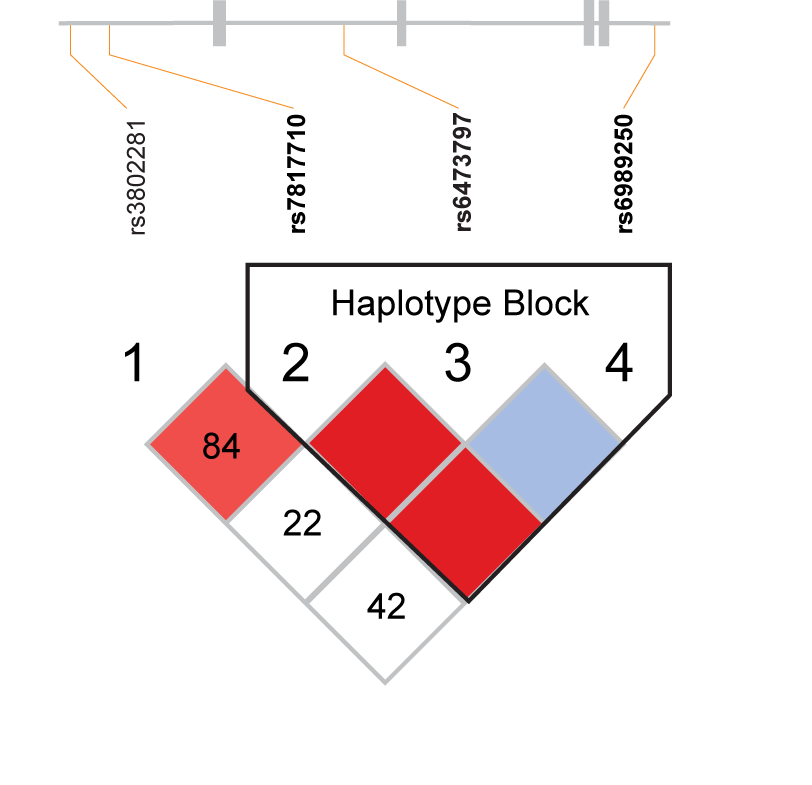


**Supplemental Figure 2 Linkage disequilibrium results in experiment 2 and 3 cohorts**

Linkage disequilibrium results for all SNPs in Hardy-Weinberg Equilibrium (rs3802281, rs7817710, rs6473797, and rs6989250) generated using Haploview software. Numbers represent *D*’ value, with colors varying by *D*’ and LOD as follows: *D*’<1, LOD<2=White; *D*’<1, LOD≥2=pink-to-red shades; *D*’=1, LOD<2=blue; *D*’=1, LOD≥2=dark red. Blocks were defined by the Four Gamete Rule.

**Supplemental Figure 2a** Linkage disequilibrium in experiment 2 cohort


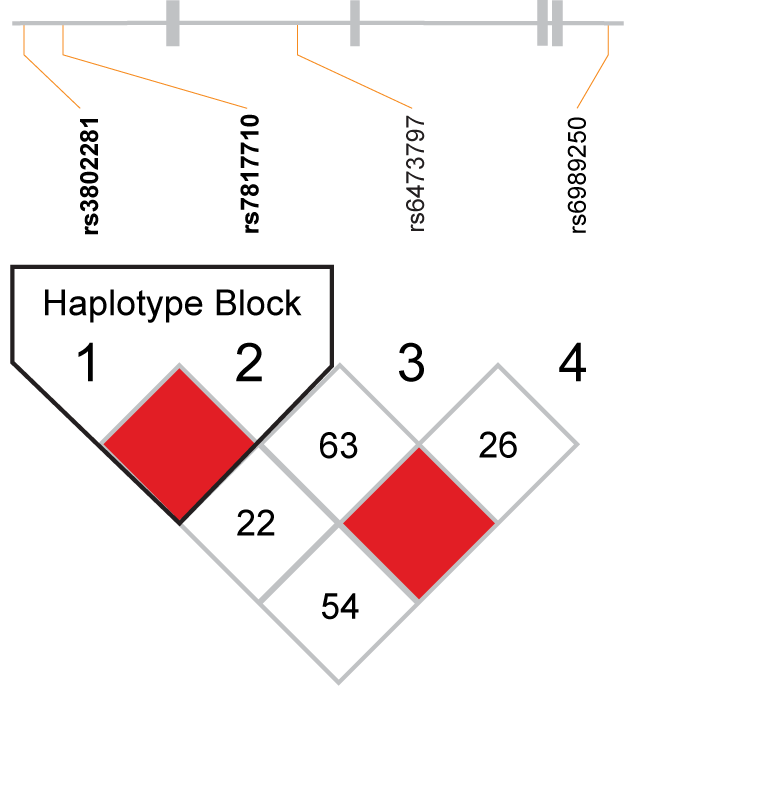


**Supplemental Figure 2b** Linkage disequilibrium in experiment 3 cohort


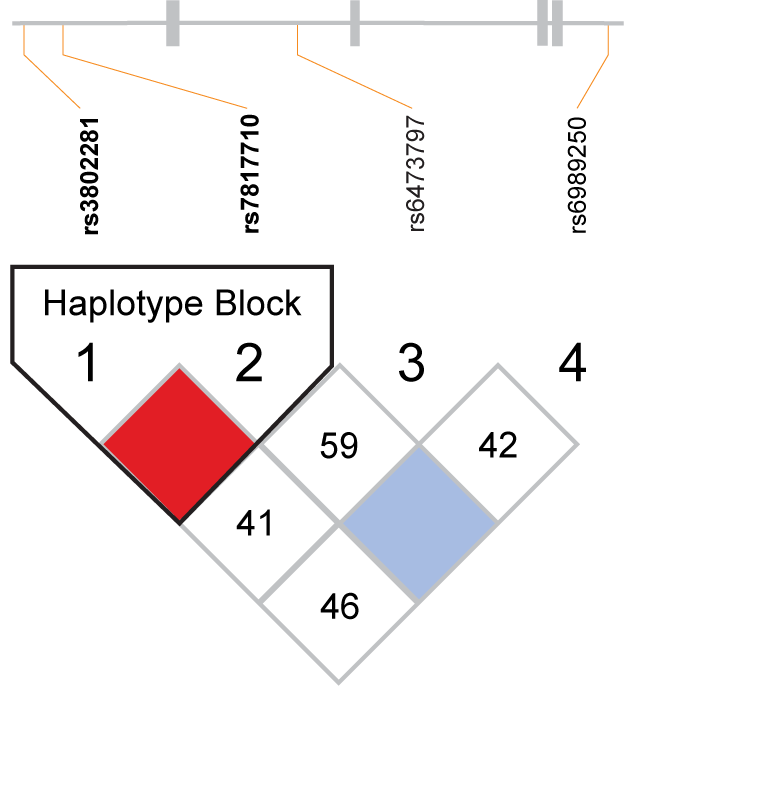

Supplement: Supplementary file 1 [file Supplementaryfile1.docx]
